# Successful endotracheal intervention for primary tracheal acinic cell carcinoma: A case report and literature review

**DOI:** 10.1097/MD.0000000000037033

**Published:** 2024-02-09

**Authors:** Shuhui Huang, Xinru Peng, Hailong Li, Jiale Zhao, Jia Hou

**Affiliations:** a Ningxia Medical University, Ningxia, China; b Department of Respiratory Medicine, Ningxia Hospital of Integrated Traditional Chinese and Western Medicine, Ningxia, China; c Department of Respiratory and Critical Care Medicine, General Hospital of Ningxia Medical University, Ningxia, China.

**Keywords:** endotracheal intervention, rigid bronchoscopy, tracheal acinic cell carcinoma

## Abstract

**Introduction:**

Primary tracheal acinic cell carcinoma (ACC) is an exceptionally rare malignancy, posing challenges in understanding its clinical behavior and optimal management. Surgical resection has traditionally been the primary treatment modality, but we present a compelling case of tracheal ACC managed with endotracheal intervention, challenging conventional approaches.

**Patient Concerns:**

A 53-year-old woman presented with shortness of breath, cough, and hemoptysis. Enhanced computed tomography revealed an obstructive tracheal lesion, leading to her referral for further assessment.

**Diagnosis:**

Microscopic evaluation, immunohistochemistry, and clinical assessments confirmed primary tracheal ACC, an exceedingly rare condition with limited clinical insights.

**Interventions:**

We utilized rigid bronchoscopy to perform endotracheal intervention, successfully resecting the tumor and restoring tracheal patency. Postoperatively, the patient received no radiotherapy or chemotherapy.

**Outcomes:**

The patient achieved complete recovery, with 24-month follow-up examinations indicating no recurrence or metastatic disease. Only minimal scar tissue remained at the resection site.

**Conclusion:**

This case demonstrates the potential of endotracheal intervention as a curative approach for primary tracheal ACC, minimizing invasiveness and preserving tracheal function. Collaborative research efforts and extensive case reporting are crucial for advancing our understanding of this rare malignancy and optimizing treatment strategies for improved patient outcomes.

## 1. Introduction

Acinic cell carcinoma (ACC) is a neoplastic entity predominantly associated with the major salivary glands.^[1]^ Nonetheless, the occurrence of primary ACC within the trachea represents an exceedingly rare manifestation of this malignancy, with limited information available regarding its clinical behavior and optimal management strategies.^[2–4]^ Current consensus underscores the necessity of surgical intervention as the primary treatment modality for patients diagnosed with this unusual condition. In this manuscript, we present a compelling case of tracheal ACC, offering valuable insights from the perspective of interventional pulmonology, which remains relatively unexplored in the context of this rare malignancy. Our report delves into the successful utilization of endotracheal intervention as an alternative therapeutic approach, resulting in the complete resection of the tumor, challenging the conventional reliance on surgical resection in such cases. This case serves as an essential contribution to the evolving understanding of primary tracheal ACC, a condition characterized by limited clinical insights and underscores the potential of endotracheal intervention as a viable treatment avenue.

## 2. Case report

**Figure 1.**
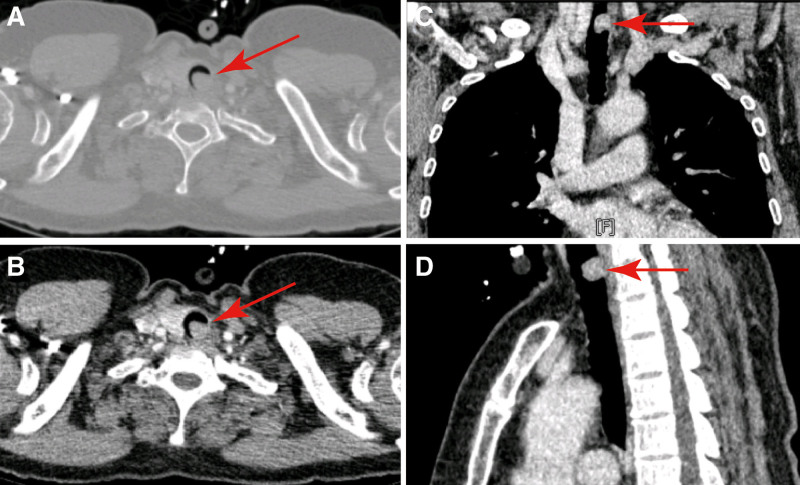
CT scan shown a 1.34 × 1.23 cm sized protruding mass in the left posterior wall of the superior trachea resulting in a 90% obstruction of trachea lumen. (A) Transverse CT lung window setting images; (B) post-contrast enhanced image; (C) coronal post-contrast enhanced image; and (D) sagittal post-contrast enhanced image.

A 53-year-old woman presented with shortness of breath, cough, and hemoptysis 2 months prior to her current presentation. Notably, she did not experience fever, chest pain, or any other relevant symptoms during this time. Six days prior to her admission to our hospital, she had been hospitalized elsewhere and received pharmaceutical treatment aimed at relieving her symptoms. This treatment included “piperacillin sulbactam” and measures to address coughing and hemostasis. Regrettably, these interventions did not yield significant relief. Subsequently, she was referred to our hospital for further medical assessment. A physical examination upon admission showed blood pressure of 130/80 mm Hg and a regular pulse of 110 beats/min. Lung auscultation revealed inspiratory wheezes in the larynx and both upper lungs. Enhanced computed tomography unveiled a soft tissue density nodule shadow within the tracheal cavity. Measuring approximately 1.34 × 1.23 cm with a computed tomography value of around 60 Hounsfield units, the lesion almost completely occluded the tracheal lumen while maintaining the integrity of the tracheal ring (Fig. 1). The remaining bronchi exhibited no obstruction, expansion, or stenosis. Importantly, there was no enlargement of the tracheobronchial or subcarinal lymph nodes. The preliminary diagnosis pointed to an intratracheal mass, leading to the subsequent resection of the lesion using a rigid bronchoscope.

**Figure 2.**
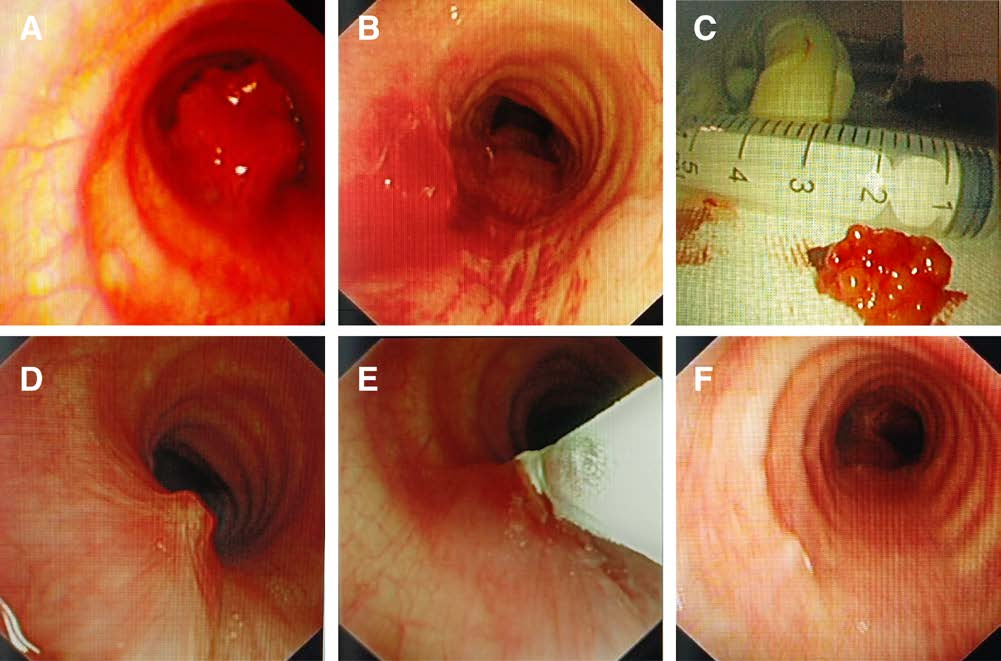
Bronchoscopic perspectives of tracheal lesions. (A) Initial view of the tracheal lesion, causing an approximately 80% obstruction. (B) Post-resection image following the removal of the tumor. (C) The tumor excised via tracheoscopy. (D) Recurrence of the tumor in situ 1 yr later. (E) Bronchoscopic images during cryotherapy treatment for tracheal luminal tumors. (F) Tracheal appearance 24 mo after resection using rigid bronchoscopy.

During the interventional bronchoscopy, we observed a tumor mass protruding into the tracheal lumen, situated 3 cm below the glottis, where the lumen appeared notably narrow (Fig. 2A). The tumor exhibited a rough and irregular surface and displayed a robust blood supply. We successfully excised the tumor using an electric snare, electrotomy, cordectomy, and an argon knife. Patency of the trachea was increased to almost normal (Fig. 2B–E). Postoperatively, the patient did not undergo radiotherapy or chemotherapy and made a complete recovery following the interventional procedure. Since then, she has undergone regular follow-up examinations every 6 months, including one sessions of bronchoscopy cryotherapy over the 2 years of follow-up to date. Notably, the 24-month follow-up examination showed no signs of recurrence or other metastatic diseases. Bronchoscopically, only a minimal amount of scar tissue remains at the site of the original lesion (Fig. 2F). The patient provided written informed consent for the publication of this case report and the inclusion of accompanying images.

## 3. Immunohistochemical and histochemical results

**Figure 3.**
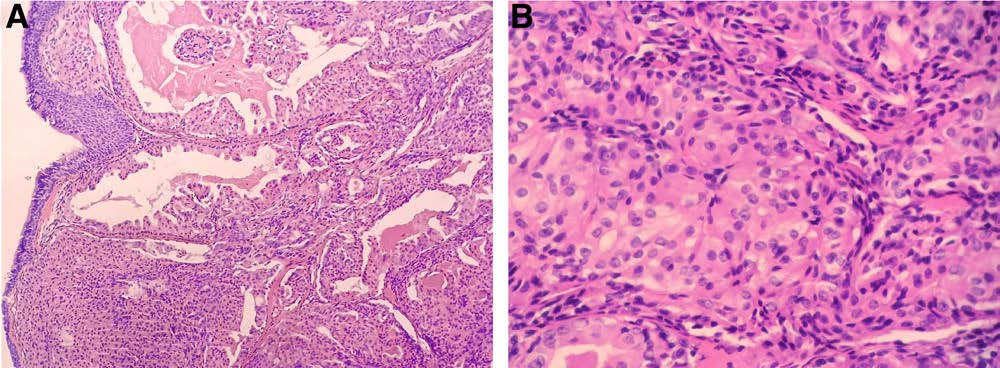
Histological features of primary ACC of the trachea. (A) Tumor cells exhibit solid nests, acinar or microcystic structures, with minimal fibrous stroma and slender, abundant blood vessels (H&E staining, 100 × magnification). (B) Tumor cells displayed relatively uniform round or polygonal shapes, with abundant granular eosinophilic or clear cytoplasm. Some tumor cells also had mucinous secretions. Cell nuclei were small, uniform, and bland with fine chromatin. They exhibit minimal pleomorphism and low mitotic activity (H&E staining, 200 × magnification). ACC = acinic cell carcinoma.

**Figure 4.**
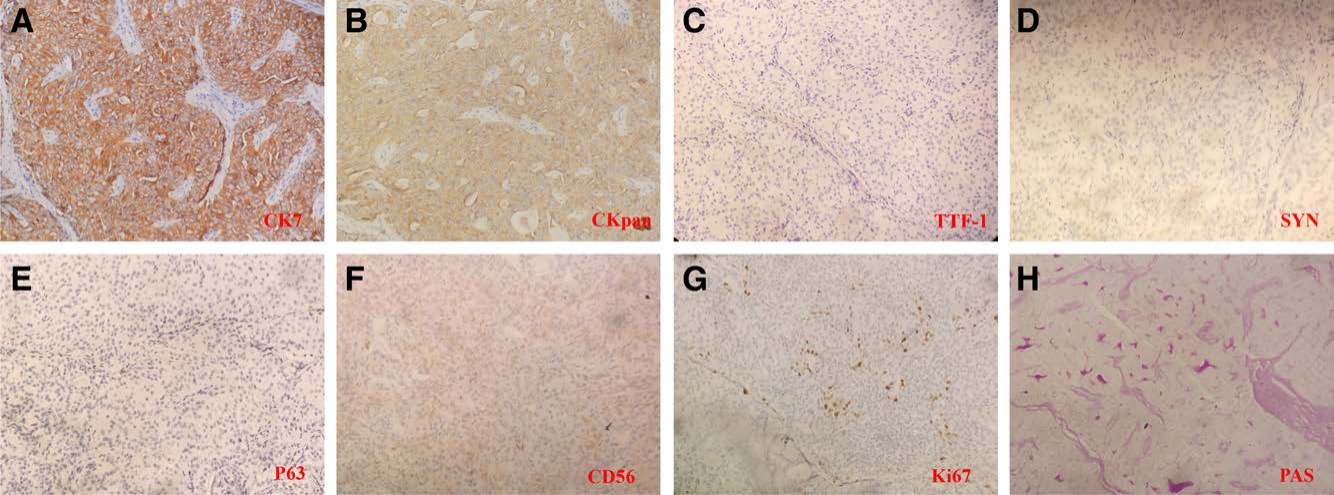
Immunohistochemical results of primary ACC of the trachea. (A and B) The neoplastic cells were positive for CK7 and CKpan (100 × magnification). (C) The percentage of proliferating cells that stained by Ki-67 was 5%. (D–F) They were negative for CD56, SYN, TTF-1, and p63. ACC = acinic cell carcinoma, TTF-1 = thyroid transcription factor-1.

Microscopically, all pulmonary ACCs were situated beneath the bronchial epithelium without morphological transitions. The nuclei of the tumor cells displayed various shapes—round, oval, or mildly polygonal—with visible nucleoli. The cytoplasm exhibited eosinophilic granularity, clarity, or vacuolation (Fig. 3). The tumor cells were positive for CKpan and CK7, while being negative for CgA, Syn, thyroid transcription factor-1 (TTF-1), P63, and P40. CD56 exhibited slight positivity. The Ki-67 staining revealed a proliferation rate of 5%. PAS staining showed positivity in a few mucous cells and the amorphous secretions within the microcystic structure (Fig. 4). No primary lesions were detected in the salivary glands, leading to the diagnosis of primary tracheal ACC.

## 4. Discussion

Primary ACC of the trachea is an exceptionally rare malignancy, with only a handful of documented cases in medical literature. Due to its scarcity, a comprehensive understanding of the clinical characteristics, optimal treatment approaches, and long-term outcomes associated with this unique tracheal cancer remains limited. In this section, we aim to place our case within the context of existing research, highlighting key findings and challenges encountered in managing primary tracheal ACC.

Based on data regarding ACC affecting the major salivary glands, this tumor is typically classified as a low-grade malignant growth, boasting a 90% 5-year survival rate and a 12% rate of distant metastasis. It worth noting that local recurrence occurs at approximately 30%, often manifesting more than a decade following the initial surgical resection.^[5,6]^ Primary ACC in the trachea and lungs typically presents as an endobronchial mass, originating from submucosal glands.^[7]^ These salivary gland-type tumors of the lungs are exceedingly rare, accounting for <1% of all pulmonary tumors.^[8]^ Primary pulmonary ACC is classified as a low-grade malignancy, typically appearing as an isolated tumor near or in close proximity to the bronchus. ACC appears to arise from the terminal salivary gland tubules or intercalated duct cells.^[9,10]^ While it was first described in 1892 by Nasse, its recognition as a distinct pathological entity came in the 1950s when Foote, Frazell, and Godwin et al delineated its characteristics.^[11]^ In 1991, the World Health Organization replaced the term “tumor” with “carcinoma” for the purpose of recognition, prognosis, and treatment. In 1972, Fechner et al documented the initial case of primary ACC in the lung.^[12]^ Since then, only 32 cases of primary pulmonary ACC have been reported, with just 4 documented in the trachea.^[13–16]^

Primary tracheal tumors, including ACC, account for <0.1% of all respiratory tract neoplasms, with ACC being a rare histological subtype within this category.^[17]^ Available epidemiological data indicate a slight female predominance,^[18]^ similar to what we observed in our case. No ethnic or racial predilection showed an association with ACC. However, specific risk factors or etiological factors have yet to be conclusively identified. The age at presentation varies widely, with cases reported in individuals spanning from adolescence to late adulthood.^[19]^

ACC belongs to the family of adenocarcinomas. Differentiating primary ACC of the lung or trachea from other primary or metastatic lung tumors is imperative.^[20,21]^ A comprehensive examination of medical records and clinical assessments can help identify pulmonary metastatic lesions originating from primary ACC of the head and neck. Several tumors sharing similar histological characteristics must be differentiated from primary lung ACC, such as lung adenocarcinoma, squamous cell carcinoma, mucoepidermoid carcinoma, carcinoid tumors, and metastatic renal clear cell carcinoma. Notably, lung adenocarcinomas and squamous cell carcinomas exhibit distinct nuclear atypia and histological or immunohistochemical evidence of glandular or squamous differentiation. Immunohistochemical markers such as TTF-1, Napsin A, P63, P40, CgA, Syn, CD56, and specific cellular features aid in this differentiation.^[22]^ TTF-1 and Napsin A staining typically yield positive results for adenocarcinoma cells, while P63 and P40 are typically positive for squamous carcinoma cells. Among neuroendocrine markers, CgA, Syn, and CD56 are the most commonly used, with CgA being the most specific and CD56 the most sensitive, albeit lacking in specificity.^[23]^ Mucinous epidermoid carcinomas typically demonstrate 3 distinct cell types: mucus-secreting cells, intermediate cells, and squamous cells, with the latter 2 typically testing positive for P63 and P40. In contrast, ACC-associated cells are negative for these markers.^[22]^ Ki-67, a marker of cell proliferation linked to mitosis, plays a vital role in assessing tumor growth rate, tissue differentiation, and chemotherapy sensitivity, with a Ki-67 ≤ 5% indicating low malignancy potential.^[24]^

The primary treatment approach for primary tracheal ACC is surgical resection, tailored to factors such as tumor size, location, and invasion of nearby structures.^[25]^ However, surgical resection, though effective, has drawbacks compared to endotracheal intervention therapy. It is more invasive and carries a higher potential for functional impairments. Procedures like tracheal resection and reconstruction, often necessary for surgical resection, can result in voice changes, swallowing difficulties, and the need for a tracheostomy tube. In contrast, minimally invasive endotracheal interventions aim to preserve tracheal function and minimize the risk of functional issues. This advantage aligns with the goal of enhancing the quality of life for patients with this rare tracheal malignancy.

In our case, rigid bronchoscopy allowed precise visualization and complete tumor excision, resulting in a favorable outcome. The role of adjuvant therapies, such as radiotherapy and chemotherapy, in primary tracheal ACC remains uncertain, primarily due to the rarity of the disease. Some reports suggest potential benefits in cases of advanced disease or positive surgical margins, but further research is necessary to establish clear guidelines. An interesting aspect of our case is the decision not to administer postoperative adjuvant radiotherapy or chemotherapy, which may be attributed to the limited data on their efficacy for primary ACC of the trachea. The patient excellent long-term outcome, with no signs of recurrence or metastasis at the 24-month follow-up, suggests that complete excision with endotracheal intervention can be curative in select cases.

The long-term prognosis for primary tracheal ACC poses challenges in establishing definitive conclusions due to the limited number of cases and the variability in treatment approaches. Nonetheless, the existing body of literature suggests that complete surgical excision, as achieved in our case, can yield favorable outcomes with no evidence of disease recurrence or distant metastasis during follow-up.^[26,27]^ However, it remains imperative to maintain vigilant, long-term surveillance, as late recurrences are a potential concern. Given the rarity of primary tracheal ACC, collaborative efforts and extensive case reporting are crucial for accumulating knowledge and formulating evidence-based management guidelines. Future research should prioritize elucidating the molecular characteristics of tracheal ACC, assessing the effectiveness of adjuvant therapies, and refining diagnostic and staging criteria. Additionally, comprehensive, long-term follow-up studies are essential for gaining a deeper understanding of disease behavior and the risk of late recurrences.

In summary, while primary ACC of the trachea remains a rare entity, this case contributes to the expanding body of literature on its diagnosis and management. Ongoing research and case reporting are critical to advancing our understanding of this unusual tracheal malignancy and optimizing treatment strategies for improved patient outcomes.

## Acknowledgments

The authors thank the patient for participating in this study and the medical staff involved in the patient diagnosis and treatment.

## Author contributions

**Investigation:** Jia Hou, Shuhui Huang, Hailong Li, Jiale Zhao.

**Methodology:** Xinru Peng, Hailong Li.

**Supervision:** Jia Hou.

**Writing – original draft:** Shuhui Huang, Xinru Peng.

**Writing – review & editing:** Jia Hou.
